# Observation and imitation of actions performed by humans, androids, and robots: an EMG study

**DOI:** 10.3389/fnhum.2015.00364

**Published:** 2015-06-19

**Authors:** Galit Hofree, Burcu A. Urgen, Piotr Winkielman, Ayse P. Saygin

**Affiliations:** ^1^Department of Psychology, University of California, San Diego, San Diego, CAUSA; ^2^Department of Cognitive Science, University of California, San Diego, San Diego, CAUSA; ^3^Behavioural Science Group, Warwick Business School, University of Warwick, CoventryUK; ^4^Department of Psychology, University of Social Sciences and Humanities, WarsawPoland

**Keywords:** electromyography, mirror neuron system, imitative processing, action perception, body movements, human robot interaction, social robotics, social cognition

## Abstract

Understanding others’ actions is essential for functioning in the physical and social world. In the past two decades research has shown that action perception involves the motor system, supporting theories that we understand others’ behavior via embodied motor simulation. Recently, empirical approach to action perception has been facilitated by using well-controlled artificial stimuli, such as robots. One broad question this approach can address is what aspects of similarity between the observer and the observed agent facilitate motor simulation. Since humans have evolved among other humans and animals, using artificial stimuli such as robots allows us to probe whether our social perceptual systems are specifically tuned to process other biological entities. In this study, we used humanoid robots with different degrees of human-likeness in appearance and motion along with electromyography (EMG) to measure muscle activity in participants’ arms while they either observed or imitated videos of three agents produce actions with their right arm. The agents were a Human (biological appearance and motion), a Robot (mechanical appearance and motion), and an Android (biological appearance and mechanical motion). Right arm muscle activity increased when participants imitated all agents. Increased muscle activation was found also in the stationary arm both during imitation and observation. Furthermore, muscle activity was sensitive to motion dynamics: activity was significantly stronger for imitation of the human than both mechanical agents. There was also a relationship between the dynamics of the muscle activity and motion dynamics in stimuli. Overall our data indicate that motor simulation is not limited to observation and imitation of agents with a biological appearance, but is also found for robots. However we also found sensitivity to human motion in the EMG responses. Combining data from multiple methods allows us to obtain a more complete picture of action understanding and the underlying neural computations.

## Introduction

Understanding the movements and actions of others is critical for survival in many species. For humans, this skill supports communicative and social behaviors, such as empathy, imitation, social learning, synchronization, and mentalizing (Blakemore and Decety, 2001; Brass and Heyes, 2005; Iacoboni, 2009; Hasson et al., 2012). The neural network in the human brain that supports action processing includes multiple brain areas, including neural systems related to visual processing of body form and motion, and the fronto-parietal mirror neuron system (MNS), which supports action understanding via *analysis-by-synthesis* (Rizzolatti et al., 2001; Saygin, 2012).

Although the MNS has been studied intensively in the past few decades, much remains to be specified about the functional properties of the system and the mechanisms that support action understanding. Our research aims to contribute to these goals, specifically in relation to form and motion information in the seen action stimuli. Vision researchers often describe perceptual mechanisms of phenomena of interest and functional properties of brain areas – e.g., whether there is evidence for motion direction selectivity, contrast modulation, category sensitivity (e.g., objects, faces), or retinotopy (e.g., Felleman and Essen, 1991). Although there have been studies of action processing and the MNS that manipulated visual stimulus properties such as body form and biological motion (e.g., Buccino et al., 2004; Saygin et al., 2004b; Casile et al., 2010; van Kemenade et al., 2012; Miller and Saygin, 2013), detailed manipulation of visual stimulus parameters to specify response properties of the MNS has not been as common an approach, possibly because mirror neurons are thought to encode high-level information such as action goals regardless of the specific sensory signals that transmit such information (Rizzolatti and Craighero, 2004). From a systems neuroscience perspective, however, such properties and related neural regions are important to specify (e.g., Giese and Poggio, 2003; Saygin et al., 2004a; Jastorff and Orban, 2009; Nelissen et al., 2011; Saygin, 2012). Going forward, a thorough understanding of the functional architecture of the relevant networks will be essential as a foundation for building less simplistic and more complete neuro-computational accounts of action understanding.

One way of doing this is the exploration of human behavior and brain responses in response to artificial agents, such as robots. Artificial agents can be programmed to perform actions, but offer different degrees of human-likeness and realism, and can be systematically varied on critical variables such appearance and motion (Chaminade and Hodgins, 2006; Chaminade and Cheng, 2009; Saygin et al., 2011). The use of robots in action observation and imitation tasks is also interesting from an evolutionary perspective given that the primate brain has, as far as we know, evolved without exposure to robots. Thus, studies with artificial agents can offer insights into psychological mechanisms in perception and action understanding as well as functional properties of underlying neural systems (Pelphrey et al., 2003; Nelissen et al., 2005; Chaminade et al., 2007; Shimada, 2010; Carter et al., 2011; Cross et al., 2012; Saygin et al., 2012; Urgen et al., 2013). Furthermore, developments in the field of robotics have led to the creation of hyper-realistic androids that invoke a future in which these kinds of robots will be deployed closer to humans than ever before (Coradeschi et al., 2006; Dautenhahn, 2007; Kahn et al., 2007). Artificial agents pose interesting questions for the psychology and neuroscience community, since it is not yet clear how we perceive and interact with such characters, especially those “almost-but-not-quite-human” agents that can evoke negative emotional responses according to the uncanny valley hypothesis (Ishiguro, 2006; MacDorman and Ishiguro, 2006; McDonnell et al., 2012; Saygin et al., 2012; Urgen et al., 2015). In turn, the robotics and animation fields are also interested in defining design parameters that will increase the acceptability and usability of the agents they develop, including in terms of appearance and motion (e.g., Chaminade and Hodgins, 2006; Kanda et al., 2008; Saygin et al., 2011; Riek, 2013).

Here, we focused on whether and how variations in an agent’s human-likeness in (i) appearance and (ii) motion influence basic motor processes occurring during action observation and imitation, and the implications of such findings for mechanisms of action processing. There is evidence that similarity between self and other is important for observation and imitation of others. For example, humans spontaneously mimic android and avatar emotional facial expressions (Weyers et al., 2006; Hofree et al., 2014), but such mimicry is modulated by how humanlike the agent appears to the observers (Hofree et al., 2014). In the domain of action and body movement observation, neural activity of the human MNS appears sensitive to visual and motor similarity between the observer and actor (e.g., Calvo-Merino et al., 2006; Cross et al., 2006). Neuroimaging studies with robots or avatars as experimental stimuli have also been carried out. Many of these studies reported that robot movements engage the motor system, though there are discrepancies among studies (Kilner et al., 2003; Tai et al., 2004; Chaminade and Hodgins, 2006; Chaminade et al., 2007; Gazzola et al., 2007; Oberman et al., 2007; Press et al., 2007; Shimada, 2010; Carter et al., 2011; Cross et al., 2012; Saygin et al., 2012; Urgen et al., 2013).

The link between action production and observation has also been explored in “automatic imitation” or “visuomotor priming” paradigms, where participants perform an action that is either compatible or incompatible with an observed movement (for review, see Heyes, 2011; Gowen and Poliakoff, 2012). If action observation and action production employ shared mechanisms, performing an action that is compatible with the observed action could lead to facilitation in performance. In contrast, performing an action that is incompatible with the observed action could result in an interference effect, i.e., slowing or disruption of performance. Several studies investigated such facilitation or interference effects with human actions (Craighero et al., 1996, 1998; Brass et al., 2000; Stürmer et al., 2000), including work exploring their modulation by factors such as biological form or motion (Kilner et al., 2003; Press et al., 2005; Bouquet et al., 2007; Longo et al., 2008; Crescentini et al., 2011). With robot actions, the results have not been entirely consistent, with reports of automatic imitation for robots (Press et al., 2005), or of effects only limited to biological actions or agents (Kilner et al., 2003; Kupferberg et al., 2012; Hayes et al., 2013), or of more complex interactions (e.g., Chaminade et al., 2005; Liepelt et al., 2010). More specific manipulations of temporal and spatial parameters in these paradigms appear promising for unifying the results (Christensen et al., 2011).

**FIGURE 1:**
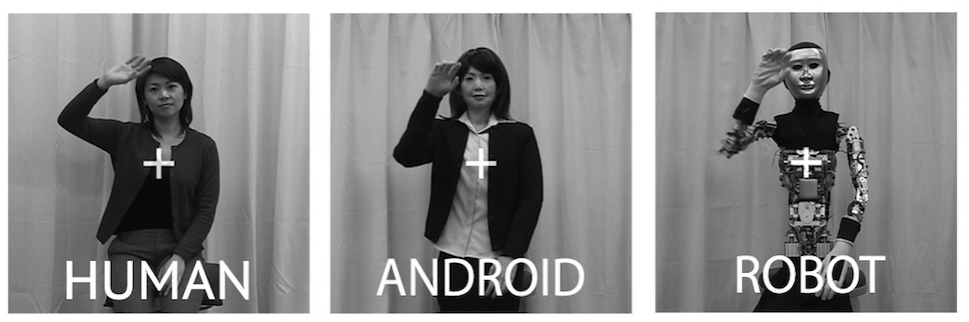
**Example stills from the videos used as stimuli in the experiment.** Here, we can see Repilee Q2 in both ‘Robot’ and ‘Android’ form, and the human ‘master’ it was modeled on. These three types of videos enabled us to compare across *Human Appearance* and *Human Motion*.

In most previous studies of action observation and imitation that used robots, the stimuli were usually not systematically varied in terms of visual properties such as appearance and motion. Robots with different characteristics were used and compared with humans, which prevents us from reaching conclusions regarding specific visual aspects that may modulate the responses. To overcome these limitations, we collaborated with a robotics lab and developed a well-controlled stimulus set of upper body movements performed by three agents, and manipulated the appearance and motion of the agents (see **Figure 1**, Materials and Methods, and Saygin et al., 2012). These stimuli consist of actions of three agents: a Human, a mechanical-looking humanoid Robot, and a human-looking robot (Android). The ‘Android’ and ‘Robot’ are actually two visually different versions of the same agent (the humanoid robot Repliee Q2), while the ‘Human’ in the videos is the woman whose appearance the Android was modeled after. These visual differences create several comparisons of interest. The Human and Android are very similar in appearance (both biological) but differ in motion dynamics. The Android and Robot are matched in their motion dynamics, but differ in appearance. The Human and Robot, although differing from each other in both appearance and motion, share the feature of having congruence between these factors (both biological and both mechanical, respectively), whereas the android features a mismatch (biological appearance, mechanical motion). These stimuli thus enable us to examine in a controlled fashion how these distinctions might influence action observation and imitation.

Using this special stimulus database, we recently performed behavioral, functional magnetic resonance imaging (fMRI) and EEG studies, demonstrating that both the appearance and the congruency of features (i.e., compatible appearance and motion) can influence action processing – but that this modulation varies depending on the behavioral task, across brain regions, and in different time scales (Saygin et al., 2012; Li et al., 2015; Urgen et al., 2015). These and similar studies described above demonstrate the utility of systematically manipulating the visual parameters of sensory input with artificial stimuli (e.g., robots) in studying human action processing and the MNS. In addition, they highlight the importance of using multiple complementary methods of inquiry. In the present study, we added to this work by using electromyography (EMG), and also extended the experimental paradigm to include explicit action imitation in addition to observation.

Although much of the research on MNS in relation to action observation and imitation has focused on regions in premotor and parietal cortex (Rizzolatti and Craighero, 2004; Iacoboni, 2009; Molenberghs et al., 2009), primary motor cortex is also involved in action perception (Borroni and Baldissera, 2008; Hari et al., 2014). Action observation, imitation, and imagery have been linked to primary motor cortex in studies with TMS in combination with motor evoked potentials (MEP, e.g., Fadiga et al., 1995, 2005), fMRI (e.g., Iacoboni et al., 1999), EEG/MEG (e.g., Hari et al., 1998; Järveläinen et al., 2001; Caetano et al., 2007; Kilner and Frith, 2007; Kilner et al., 2009; Neuper et al., 2009), intracranial recordings (Mukamel et al., 2010) and occasionally with EMG (Leighton et al., 2010). There are strong reciprocal connections and modulatory influences between premotor cortex (specifically area F5, which contains mirror neurons) and primary motor cortex (Iacoboni et al., 1999; Shimazu et al., 2004). Although EMG is not a direct measure of cortical motor activity and can be susceptible to other non-cortical influences, measuring the activity of actual muscles enables us to obtain a reasonable index of primary motor cortex activity associated with the peripheral motor system (Santucci et al., 2005; Kalaska, 2009; Churchland et al., 2012). In the current study, we recorded muscle activity of the arms of human subjects during observation and imitation of arm actions. Using human and non-human agents as stimuli, we explored how features of the observed agent might modulate EMG – specifically, humanlike motion or humanlike appearance.

Besides adding a new methodology with different strengths to study the functional properties of the MNS, the use of EMG could also help bridge the work on action observation and imitation with the work on facial mimicry. EMG has long been used in studying spontaneous facial mimicry, an automatic process that occurs without explicit instruction (Dimberg, 1982; Carr and Winkielman, 2014). Although, as mentioned above, automatic imitation is thought to occur also for bodily movements and actions (Heyes, 2011), the use of EMG in this field has been rare. Berger and Hadley (1975) found increased arm and lip EMG response during observation of non-emotional actions. Moody and McIntosh (2011) replicated these findings for facial but not arm muscles. Furthermore, since EMG is a continuous measure of muscle activity, it creates the potential for linking the dynamics of the motor responses with those of the visual action stimuli, which by nature are temporally unfolding. In this way, we can assess differences in synchronization between the participant and the observed agent both for the observation and imitation conditions, as was done in a recent study that used EMG to examine synchronization of facial expressions between human participants and a robot (Hofree et al., 2014). Finally, EMG allows us to readily investigate the peripheral motor activity in both arms – left and right – as participants observe or imitate an agent performing an action with one arm. This enables us to explore whether there is muscle activity in the arm that is not directly performing an action, and the lateralization of responses during action observation and imitation (Aziz-Zadeh et al., 2006; Franz et al., 2007; Kilner et al., 2009; Cross et al., 2013).

## Materials and Methods

### Participants

Forty-three University of California, San Diego undergraduates were recruited. Participants had normal or corrected-to-normal vision and were right-handed and received course credit. The research protocol was approved by the University of California, San Diego Institutional Review Board. Written informed consent was obtained from all subjects. Unfortunately, data from ten subjects could not be used due to software or equipment errors, and data from six could not be used because they did not follow instructions (e.g., making arm movements during periods they were supposed to remain still). Note, however, that the final sample size of twenty-seven participants is typical for an EMG study (e.g., Hofree et al., 2014). Those participants were 18–22 years of age; 17 were female.

### Visual Stimuli

Stimuli were 2-s video clips of upper body actions performed by the state-of-the art humanoid robot Repliee Q2 and by the human ‘master’ after whom it was modeled (**Figure 1**). Repliee Q2 performed each action in two different appearance conditions: in the Android condition, Repliee Q2 appeared as is, in a highly humanlike appearance. In the Robot condition, Repliee Q2 appeared after we stripped off or covered the elements that aimed to make the agent highly humanlike in appearance (**Figure 1**). We refer to these conditions as Android and Robot, respectively, even though they were in fact the same physical robot performing the very same pre-programmed movements.

The Robot and Android conditions differed only in their appearance, with Android featuring a humanlike appearance and the Robot featuring a non-human, mechanical appearance. Crucially, the kinematics of the movement for the Android and Robot conditions were identical, since, as mentioned, they were actually the same machine. For the Human condition, the female adult whose face was used in constructing Repliee Q2 (the ‘master’ of the android) was asked to watch each of the Repliee Q2’s actions and then perform the same action naturally. Thus these videos were comparable in appearance and action to the Android version of Repliee Q2, but differed in the motion and timing dynamics of the actions. Due to inherent limitations of the robot we worked with, as well as human anatomy, we did not have the fourth condition that would have made our experimental design 2 (*Motion*) × 2 (*Appearance*): an agent with an appearance that is identical to our Robot condition but with human motion was simply not possible to generate with the present stimulus set. Therefore, even though there are three levels of the factor *Agent*, the omnibus analysis of variance (ANOVA) does not directly correspond to the hypotheses we are testing (which are reflected in the very design of the stimuli) concerning agent appearance and agent motion (see Saygin et al., 2012; Urgen et al., 2013 and Data Reduction and Analysis).

The three agents’ actions were videotaped in the same room and with the same background, lighting, position and camera, yielding a well-controlled set of stimuli. A total of eight actions per actor were used in this study: drinking water from a cup, picking up a piece of paper from a table, grasping a tube of hand lotion, wiping a table with a cloth, waving hand, nudging, turning to look at something, and introducing self (a small Japanese head bow, with the arm raised to the chest). All except the turning action were used in the EMG experiment phase; the turning action was used in the rating phase preceding and following the experiment phase. In all videos, the agent executed arm movements with the right hand. Videos were converted to grayscale and cropped at 400 × 400 pixels, with a semi-transparent white fixation cross superimposed at the center. The videos were edited such that movement started right at the beginning of the video. We extended the videos’ duration to 5 s by freezing the last frame for 3 μs, so that we could record EMG responses for a full 5 s since responses to dynamic actions can take up to 5 s to offset. Further details of the agents and the action videos are reported in previous publications (Saygin and Stadler, 2012; Saygin et al., 2012; Urgen et al., 2013; Li et al., 2015).

### Procedure

Participants sat comfortably 2 feet in front of a computer screen. Electrodes were affixed to the left side of their face and to their two arms. They were asked to place their arms in their lap. They were instructed to sit calmly, keep still, and follow the instructions on the computer screen.

Before beginning the EMG experiment, participants were briefed that they would be viewing videos of three agents. They were told explicitly whether each agent was human or a robot (cf. Saygin et al., 2012; Urgen et al., 2013). Participants then viewed a video of each agent making a turning movement (looking to the right while seated), and were asked to provide subjective ratings on several attributes (e.g., *Human-likeness* or *Comfort*, see Supplementary Materials 1.1). The presentation of the turning videos and acquisition of ratings were repeated again at the end of the main experiment. The rating data are included in Supplementary Materials 2.1. These, along with the facial EMG activity we measured, were intended to serve as measures of affective responses to help address alternative explanations for our results.

The main experiment was modeled after a classical imitation paradigm (Dimberg, 1982; Hofree et al., 2014). In each trial of the experimental phase, participants were presented with a 5-s blank screen with a fixation cross, followed by an action stimulus. As mentioned, in each video, the agent’s movement started at the onset of the movie. Once the video clip was played, the last frame was kept visible on the screen such that there was a 5-s period of visual stimulus and EMG recording for the trial. There were two task conditions administered in different blocks: an *Observation* block and an *Imitation* block. The *Imitation* and *Observation* blocks were identical except for the instructions given at the beginning of the block (i.e., the subject’s task). In the *Observation* condition, participants were instructed to simply view videos of the three agents whilst remaining still. In the *Imitation* condition they were instructed to imitate the action they saw in the video (“try and make the same action as the agent,” modeled after Dimberg, 1982). As mentioned earlier, all participants were right-handed, and the actions in the stimuli were also performed right-handed. It is well-established that adults show a very strong tendency to imitate with the same effector(s) as the observed actor (anatomical imitation, e.g., Koski et al., 2003; Franz et al., 2007), even though this is more difficult and error prone (Press et al., 2009). We therefore expected participants to imitate the movements with their right hand (see EMG Results and Supplementary Materials 1.2.2 for a control analysis). Overall, The *Imitation* block always followed the *Observation* block, in order to avoid potential expectations to imitate during the *Observation* condition (Cross and Iacoboni, 2014). In each condition, participants were presented with a random order of the three agents performing each of the seven actions six times, with a total of 126 trials per block.

### Electromyography

#### Data Acquisition

A pilot study was conducted in order to determine the arm muscle best suited for recording responses for the present stimuli. Electrodes were placed over the bicep brachii, the flexor carpi radialis and the brachioradialis muscles of a participant (member of the lab). EMG was recorded while the assistant conducted the *Imitation* block of the experiment. Based on these data, we determined that the bicep brachii was the best candidate for the actions in this experiment.

Arm EMG was recorded by pairs of 1-cm (4-cm diameter) electrodes placed over the bicep brachii muscle of each arm. The first electrode was placed in the center of the muscle, and the second was placed a collar width (~2 cm) directly below the first electrode. Facial EMG was measured by pairs of 1-cm (2.5 cm square) electrodes on the left side of the face, over the regions of zygomaticus major (cheek) and corrugator supercilii (brow), according to EMG processing standards (Tassinary and Cacioppo, 2000). For the zygomaticus major muscle, the first electrode was placed in the middle of an imaginary line between the lip corner at rest, and the point where the jaws meet (approximately near the ear lobe), the second electrode a collar width (~1 cm) posterior to the first. For the corrugator supercilli muscle, the first electrode was placed right above the left eyebrow, on an invisible vertical line from the inner corner of the eye up, the second a collar width posterior to the first (following the eyebrow arch).

AcqKnowledge software (Biopac Systems, Goleta, CA, USA) along with Biopac (Biopac Systems, Goleta, CA, USA) was used to acquire the EMG signal. The amplified EMG signals were filtered online with a low-pass of 500 Hz and a high-pass of 10 Hz, sampled at a rate of 2000 Hz, and then integrated and rectified using Mindware EMG software, version 2.52 (MindWare Technologies Ltd., Gahanna, OH, USA).

#### Data Reduction and Analysis

Data were analyzed using Matlab (version R2012b, The Mathworks, Natick, MA, USA), JMP (version 10, SAS Institute Inc., Cary, NC, USA), and SPSS (version 19, IBM Corporation, Armonk, NY, USA). Data were first averaged in 500 ms intervals across a trial (i.e., 10 data points for a 5 s trial). Extreme values (values greater than 3 SD away from the mean) were excluded from the analysis. Next, data were standardized within participant and within each muscle, using as baseline the minimum value in the 2000 ms interval before each trial, with a sliding window to smooth baseline values over trials (this technique helped remove any noisy EMG periods in between trials; see also Supplementary Materials 1.2.1). We calculated baseline-corrected activity for each participant and each muscle across the 5-s trial by removing the calculated baseline per trial from each data point (10 per trial). Finally, we averaged baseline corrected EMG activity within 500 ms intervals across trials for each individual, muscle, condition (observation, imitation), agent, and action.

The main experimental factors were Condition (*Observation* and *Imitation*), Arm (*Left* and *Right*), Motion (*Human* and *Non-Human Motion*), and Appearance (*Human* and *Non-Human Appearance*). As mentioned, due to technical reasons our stimuli do not correspond to a full factorial design with respect to appearance and motion (lacking the non-human appearance and human motion condition). The main effect/interaction structure of a conventional ANOVA thus does not correspond to the hypotheses being tested regarding these factors (cf. Saygin and Stadler, 2012; van Kemenade et al., 2012). Rather, our stimuli were designed to investigate effects of *Human* vs. *Non-Human Motion*, and *Human* vs. *Non-Human Appearance* (and the congruence of the two, see Saygin et al., 2012). The Human videos represent *Human Motion*, while the Android and Robot videos represent *Non-Human Motion*. The Human and Android videos both represent a *Human Appearance*, while the Robot video represents *Non-Human Appearance* (**Figure 1**). Therefore we conducted multivariate analysis of variances (MANOVAs) with these factors to explore how these features influenced our EMG dependent measures.

Below, we present the statistics and figures as described above to streamline the presentation. However, for the interested reader, we also provide both statistical analyses and figures that do not collapse the agent levels (i.e., three level Agent factor); but since no new findings or insights emerged, these are included in Supplementary Materials 2.2.1.

## Results

### EMG Results

Participants’ EMG responses to the videos were analyzed using repeated-measures MANOVA over all time points in the trial (measured in 500 ms intervals). We examined differences across Condition (*Observation* and *Imitation*), Arm muscles (*Left* and *Right*), Motion (*Human* and *Non-Human Motion*) or Appearance (*Human* and *Non-Human Appearance*), and Time (500 ms intervals across a 5 s trial, for a total of 10 time points).

#### Human vs. Non-Human Motion Comparisons

**FIGURE 2:**
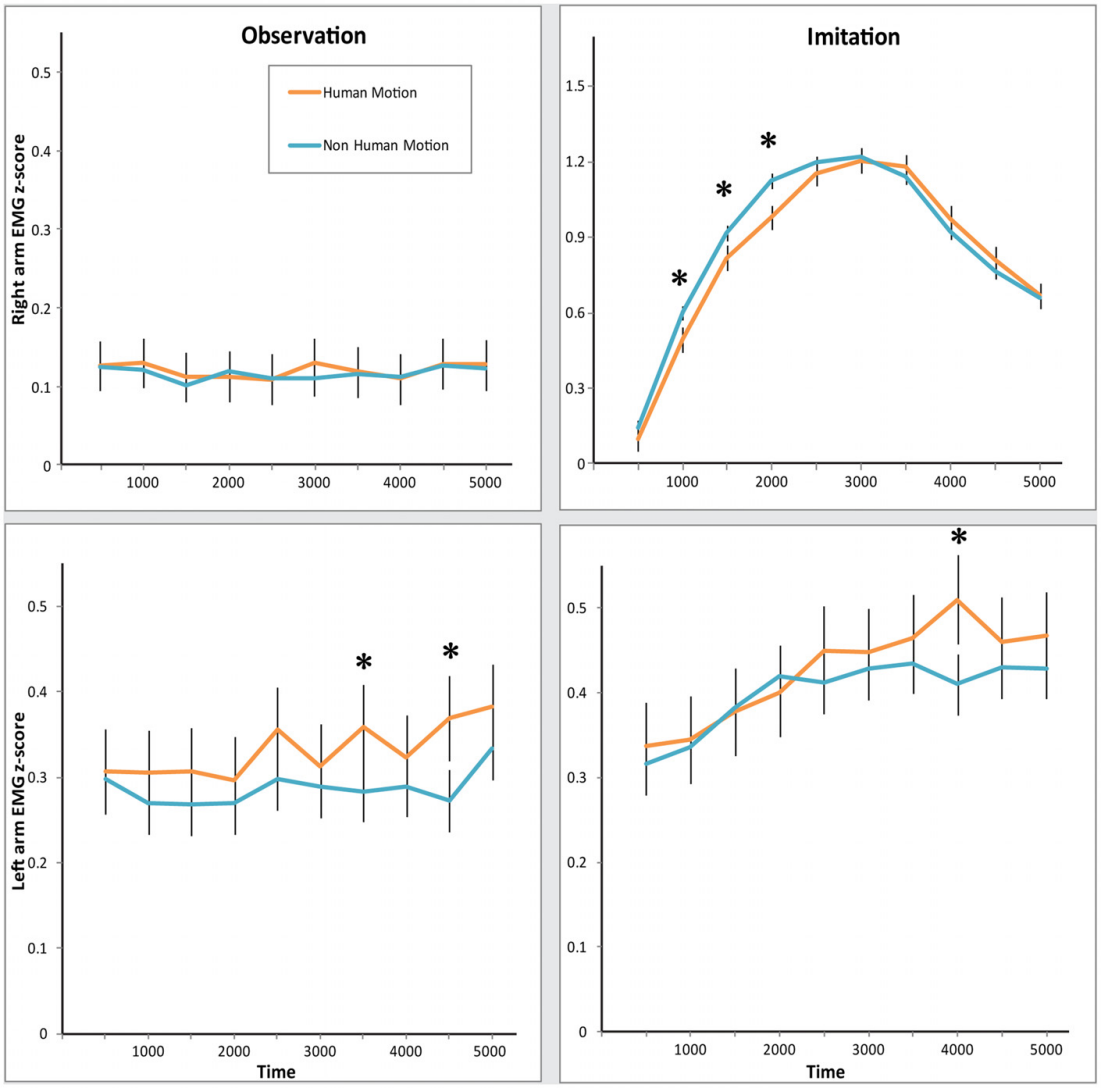
**Electromyography (EMG) response in both arms during Observation and Imitation of Human and Non-Human Motion.Top**: z-scored EMG activity in the Right arm. **Bottom**: z-scored EMG activity in the Left arm. **Left**: EMG activity during Observation condition. **Right**: EMG activity during Imitation condition. Error bars represent SEM. Asterisks denote significance across Motion, at the 0.05 level.

We ran a repeated measures MANOVA with a 2 (Condition: *Observation* vs. *Imitation*) × 2 (Arm: *Left* vs. *Right*) × 2 (Motion: *Human* vs. *Non*-*Human*) × 10 (Time) design. This MANOVA revealed several significant effects, as can be seen in **Figure 2**. First, as expected, across both arms we found more muscle activity in the *Imitation* condition than in the *Observation* condition. This is shown by the main effect of Condition [*F*(1,26) = 53.42, *p* < 0.0001, $\eta_{p}^{2}$ = 0.67], and a significant Condition × Time interaction [*F*(9,234) = 13.39, *p* < 0.0001, $\eta_{p}^{2}$ = 0.34]. Second, there was more overall muscle activity in the *Right* (dominant) arm, than in the *Left* arm, as revealed by the main effect of Arm [*F*(1,26) = 4.72, *p* = 0.04, $\eta_{p}^{2}$ = 0.15], and the Arm × Time interaction [*F*(9,234) = 11.70, *p* < 0.0001, $\eta_{p}^{2}$ = 0.31].

However, most interestingly, we found evidence that muscles of the two arms responded differently across conditions, as revealed by the Condition × Arm, and Condition × Arm × Time interactions [*Condition* × *Arm: F*(1,26) = 41.41, *p* < 0.0001, $\eta_{p}^{2}$ = 0.61; *Condition* × *Arm* × *Time: F*(9,234) = 11.71, *p* < 0.0001, $\eta_{p}^{2}$ = 0.31]. Separate MANOVAs for each condition revealed that in the *Observation* condition, there was more activity in the *Left* arm than the *Right* arm [*main effect of Arm: F*(1,26) = 21.58, *p* < 0.0001, $\eta_{p}^{2}$ = 0.45; *significant Arm* × *Time interaction: F*(9,234) = 2.29, *p* = 0.02, $\eta_{p}^{2}$ = 0.62]. At the same time, there was significantly more muscle activity in the *Right* arm than the *Left* arm in the *Imitation* condition [*main effect of Arm: F*(1,26) = 18.90, *p* < 0.0001, $\eta_{p}^{2}$ = 0.42; *significant Arm* × *Time interaction: F*(9,234) = 11.95, *p* < 0.0001, $\eta_{p}^{2}$ = 0.32]. Participants appeared to respond more strongly with their *Right* arm when told to mimic the videos, but exhibited a stronger response with their *Left* arm when just observing videos.

The two arms differed in their sensitivity to *Human Motion*, as can be seen in the significant Arm × Motion interaction [*F*(1,26) = 9.92, *p* = 0.004, $\eta_{p}^{2}$ = 0.28]. MANOVAs for each arm yielded the following arm-specific effects: the *Left* arm demonstrated a significant increase in EMG amplitude in response to *Human Motion* in both conditions [*main effect of Motion: F*(1,26) = 4.22, *p* = 0.05, $\eta_{p}^{2}$ = 0.14], while the *Right* arm did not [*no significant main effect of Motion. Motion* × *Time interaction: F*(9,234) = 3.55, *p* < 0.001, $\eta_{p}^{2}$ = 0.12; *Condition* × *Motion* × *Time interaction: F*(9,234) = 4.02, *p* < 0.0001, $\eta_{p}^{2}$ = 0.13]. However, the timing of responses differed in the *Right* arm, specifically in the *Imitation* condition. As can be seen in **Figure 2** (top right panel), the EMG mimicry response of the *Right* arm was more delayed for *Human Motion* than for *Non*-*Human Motion*. *Post hoc* comparisons of *Human Motion* and *Non-Human Motion* in the early and late half of the trial demonstrate that differences between the types of motion exist only in the first half of the trial [*M*_Human_ _Motion_ = 0.73, *M*_Non-Human_ _Motion_ = 0.84, *t*(26) = 4.32, *p* < 0.001], and disappear in the second half [*M*_Human_ _Motion_ = 0.98, *M*_Non-Human_ _Motion_ = 0.95, *t*(26) = -1.03, *p* = 0.31]. This is likely due to the slight timing differences in the videos between Repliee Q2 and the Human. This effect was specific to the *Right* arm in the *Imitation* condition [hence a significant Condition × Arm × Motion × Time interaction, *F*(9,234) = 2.02, *p* = 0.04, $\eta_{p}^{2}$ = 0.07]. Since the EMG movement in the *Right* arm in this condition was much greater in magnitude than EMG responses in any other condition (this can be seen in **Figure 2** top right panel, where the y-axis scale is three times larger than the y-axes in the other panels), we believe it also drives the significant Motion × Time [*F*(9,234) = 3.42, *p* = 0.001, $\eta_{p}^{2}$ = 0.12] and Condition × Motion × Time [*F*(9,234) = 2.81, *p* = 0.004, $\eta_{p}^{2}$ = 0.10] interactions. We tested whether the delay in reaction to *Human Motion* was due to a particularly slow response by comparing the lags of the EMG waveform that correlated the highest with the waveform produced by the movement in the corresponding videos (see Synchronization Analyses: Are Observers’ and Observed Agents’ Movements Linked?). These lags did not differ significantly, suggesting that this was most likely correlated with a timing difference in the videos.

Since the Robot is Non-Human in both motion and appearance, we compared the Android and the Human, specifically testing an effect of *Human Motion* while maintaining constant *Human Appearance*. In this MANOVA, again we found a significant interaction of Condition × Arm × Motion [*F*(1,26) = 9.53, *p* = 0.005, $\eta_{p}^{2}$ = 0.27], as well as a significant Motion × Time interaction [*F*(9,234) = 2.15, *p* = 0.03, $\eta_{p}^{2}$ = 0.08], indicating that the EMG response is specifically sensitive to *Human Motion*.

#### Human vs. Non-Human Appearance Comparisons

We ran analogous MANOVAs examining whether EMG responses were sensitive to *Human Appearance*. This MANOVA was a 2 (Condition) × 2 (Arm) × 2 (Appearance) × 10 (Time) design. We observed a significant Appearance × Time interaction [*F*(9,234) = 3.30, *p* = 0.001, $\eta_{p}^{2}$ = 0.11], as well as a Condition × Arm × Appearance × Time interaction [*F*(9,234) = 2.26, *p* = 0.02, $\eta_{p}^{2}$ = 0.08]. These again seem to be driven by the delay in EMG response in the *Right* arm in the *Imitation* condition for the Human videos. For a closer comparison of *Human* vs. *Non-Human Appearance*, while holding motion constant, we compared EMG responses to the Android and Robot conditions, where the *Appearance* was varied while maintaining the same *Non-Human Motion.* Here, there was no significant effect of Appearance.

#### Synchronization Analyses: Are Observers’ and Observed Agents’ Movements Linked?

As described above and shown in **Figure 2**, we found several significant effects of Time (i.e., changes in EMG amplitude in various points of the trial), which led us to consider whether there might be a relationship between the temporal dynamics of the human EMG response and the motion dynamics over time in the visual stimuli. To explore whether people’s movements were linked to the movement of the seen agents, we ran cross-correlation analyses with the EMG data and the motion dynamics of the stimuli. The movement dynamics in the visual stimuli were extracted using an object motion-tracking algorithm (Peddireddi, 2009), representing a rough aggregate measure of the motion of the arm in each video (since no other moving objects were present). The video arm movement and the arm EMG response were compared using cross-correlation, which allowed us to determine the lag at which maximal correlation occurred between the visual movement and the time-delayed, congruent EMG activity for each Action, Agent, Condition, and Arm. We aggregated the correlations found for each subject, for the different conditions using a Fisher’s z transformation, and compared correlations for each subject across experimental factors.

**FIGURE 3:**
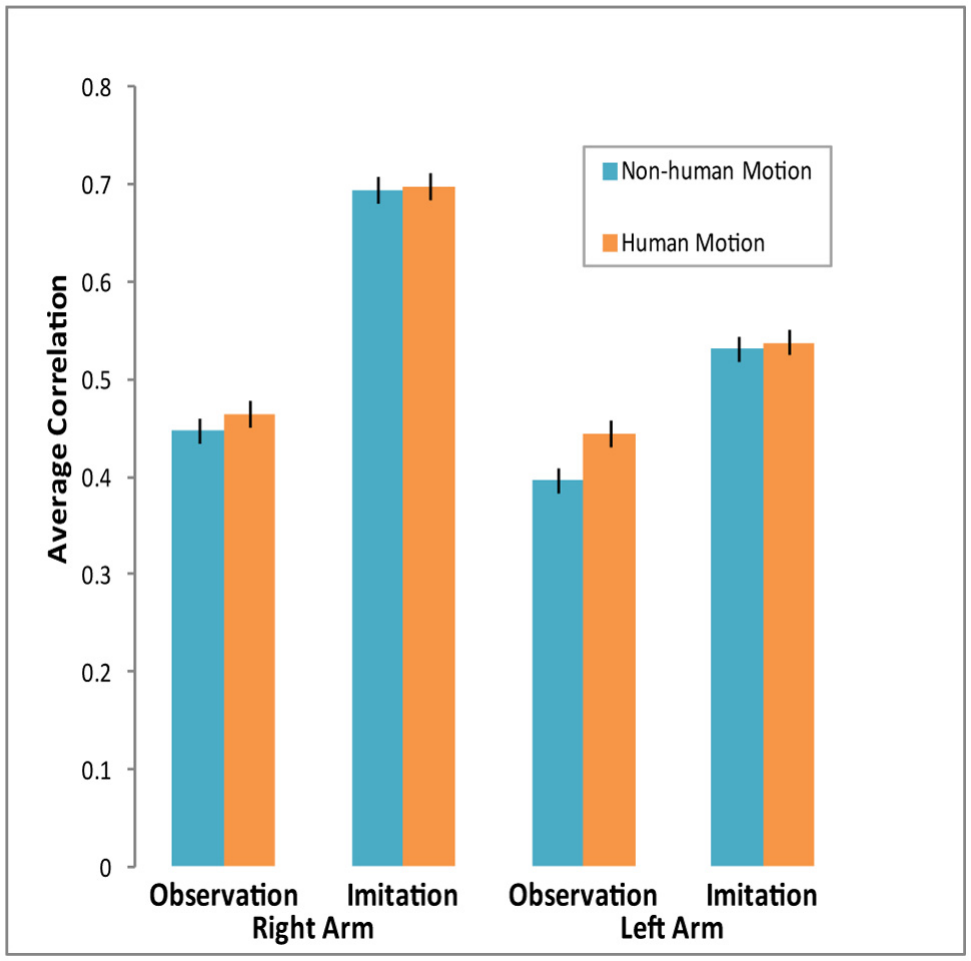
**Average correlations between EMG activity and agent arm movement in video across conditions.** Cross-correlations were computed for each individual across experimental conditions. Participants’ arm EMG activity was more strongly correlated with agent arm movement during the Imitation condition, especially for the Right arm. Arm EMG activity was also more correlated with *Human Motion* than with *Non-Human Motion*. Error bars represent SEM.

**Figure 3** shows average correlations across conditions. Though in all conditions the correlations were significant and positive, they varied across experimental conditions. A repeated-measures MANOVA over the z-transformed correlation coefficients conducted across Condition, Arm, and Motion (Human vs. Android and Robot) revealed a significant main effect of Motion. Participants’ arm EMG was more correlated with *Human Motion* than *Non-Human Motion* [*F*(1,26) = 4.45, *p* = 0.045, $\eta_{p}^{2}$ = 0.15]. We also found a main effect of Condition, with participants’ arm EMG more correlated with the observed motion in the *Imitation* than *Observation* condition [*F*(1,26) = 49.16, *p* < 0.0001, $\eta_{p}^{2}$ = 0.65]. There was also a main effect of Arm [*F*(1,26) = 18.74, *p* < 0.0001, $\eta_{p}^{2}$ = 0.42], as well as a significant Condition × Arm interaction [*F*(1,26) = 6.8, *p* = 0.02, $\eta_{p}^{2}$ = 0.21]. The *Right* arm’s EMG dynamics matched the motion in the videos much more than the Left arm, and this difference was more pronounced in the *Imitation* condition (see **Figure 3**). A similar MANOVA run with Appearance (Human and Android vs. Robot) demonstrated no effect of human appearance on correlations between EMG activity and stimulus motion.

#### Supplementary Analyses

In addition to our main factors of interests, we ran additional analyses that are provided in greater depth that may be of interest to some readers, but were not central to the study. As already mentioned, we provided the three factor analyses as well as figures showing the three Agent conditions separately in Supplementary Materials 2.2.1. We also included therein a control to ensure that left arm EMG in the imitation condition was not contaminated by actual left-arm imitation. Although adults overwhelmingly perform anatomical imitation, we set a criterion for rejecting possible mirror imitation. The vast majority of participants clearly used their right hand based on their data. We did find four subjects for whom left hand use could not be ruled out, and excluding these participants did not change the results (see Supplementary Materials 1.2.2). Thus, there is no clear indication of mirror imitation, nor does it appear that the pattern found for the left arm is an artifact of some individuals imitating with the left arm.

We also include in Supplementary Materials results of analyses that also include facial EMG data and Gender (see 2.2.3 and 2.2.4, respectively). From the analyses with Gender as a factor, we observed that *Human Motion* produced a greater effect on EMG of male subjects during the *Imitation* condition as compared with females, and that females demonstrated more *Right Arm* activity during the *Observation* condition than males. Our key findings from the facial EMG analyses were that zygomaticus activity was greater in response to *Human Motion* and *Human Appearance* than to *Non-Human Motion* and *Appearance*, during the *Imitation* condition. On the other hand, corrugator activity increased in response to *Human Motion* in the *Observation* condition. These analyses are provided for the interested reader, but given our study was not designed specifically to explore these issues, they should be considered preliminary observations.

## Discussion

The initial discovery of mirror neurons in the macaque area F5 and evidence for the involvement of motor brain regions in action perception elicited great enthusiasm (Gallese et al., 1996; Rizzolatti et al., 1996; Fadiga et al., 2005). In the following years, the MNS received intense interest and focus from neuroscientists (Rizzolatti et al., 2001; Rizzolatti and Craighero, 2004), and more broadly contributed to the resurgence of the “embodied cognition” framework (Barsalou, 1999, 2008; Wilson, 2002; Niedenthal, 2007; Grafton, 2009; Winkielman et al., 2015), echoes of which were present decades earlier in the works of prominent psychologists such as James, Gibson, and Piaget (Prinz, 1987). MNS has been proposed as a potential evolutionary and neural basis of many essential human abilities such as empathy, theory of mind, learning, and language (Rizzolatti and Craighero, 2004; Arbib, 2005; Iacoboni, 2009), and has been linked to disorders affecting social and communicative functions such as autism (Iacoboni and Dapretto, 2006). Some embraced MNS as the basis for these functions and more (e.g., “neurons that shaped civilization,” Ramachandran, 2012), others were concerned that the explanatory powers of the MNS were exaggerated (e.g., “the most hyped concept in neuroscience,” Jarrett, 2012). The importance or even the existence of the system, and implications on social functioning and development became a matter of debate (Hickok, 2009) and, more importantly, of empirical investigation (e.g., Nelissen et al., 2005; Dinstein et al., 2007; Kilner et al., 2009; Lingnau et al., 2009; Mukamel et al., 2010; Hamilton, 2013; Cook et al., 2014).

In the past few years, the vast majority of researchers in the field have rejected either extreme, viewing the MNS neither as a silver bullet, nor merely as hype. Looking at the empirical data on the MNS and embodiment, and not necessarily the interpretation of said data, it is difficult to remain unconvinced that some degree of motor processing is an important and critical part of action understanding. Two decades on, the field is moving toward a more neutral framework for thinking about the MNS and embodiment, and of course, for empirical work. This research topic is part of an increasing awareness that, despite the impressive body of work that has accumulated on the topic, much remains to be specified about the MNS and the perception and imitation of actions (Kilner and Lemon, 2013; Cook et al., 2014). Among others, topics that require further research include the response properties, origins and functions of the MNS; how MNS contributes to imitation, empathy and communication; correlational vs. causal relationships between MNS and behavior; individual differences in action processing in healthy and clinical populations; the relationship between MNS and disorders of social cognition; computational mechanisms of information processing within MNS, as well as interactions with other brain areas (Brass and Heyes, 2005; Oztop et al., 2006; Kilner et al., 2007; Engel et al., 2008; Grafton, 2009; Gilaie-Dotan et al., 2011; Mcbride et al., 2012; Sasaki et al., 2012; Avenanti et al., 2013; Fleischer et al., 2013; Hamilton, 2013; Miller and Saygin, 2013; Marshall and Meltzoff, 2014; Simpson et al., 2014).

It is worth noting that research on the functional properties of MNS has been naturally dominated by neuroimaging studies, which focus on the central nervous system. However, in the context of embodied cognition, a complete characterization of the mechanisms of action observation and imitation requires consideration of the peripheral systems as well. Here, we used EMG to examine how muscle activity might be influenced by human-likeness of the agent during action observation and imitation using stimuli of actions performed by three agents: a human agent featuring humanlike appearance and motion, an android featuring humanlike appearance and non-humanlike motion, and a robot featuring non-humanlike appearance and motion.

### Artificial Agents in Cognitive Neuroscience

In terms of our understanding of functional properties of MNS and simulation theory, which posits visually perceived actions are mapped onto the viewer’s own sensorimotor neural representations, stimuli that feature artificial form or motion patterns can allow us to explore the boundary conditions for evoking motor simulation. Artificial agents such as robots can be important experimental stimuli to test such hypotheses since robots can perform recognizable actions, but can differ from biological agents in their design (Chaminade et al., 2007; Saygin and Stadler, 2012; Urgen et al., 2013).

Although there is a growing body of research that employs robots as experimental stimuli in action observation tasks, the cognitive neuroscience literature on the perception of robots has inconsistencies (Kilner et al., 2003; Chaminade and Hodgins, 2006; Chaminade et al., 2007; Gazzola et al., 2007; Oberman et al., 2007; Press et al., 2007; Saygin et al., 2012; Urgen et al., 2013). Some studies reported that perception of robot actions results in similar activity in the MNS (as compared to that for human actions), whereas others have argued that the MNS is not responsive to nonhuman actions (Tai et al., 2004). Importantly, an fMRI study found no difference between conditions in ventral premotor cortex using the same stimuli employed in the current study (Saygin et al., 2012). In addition, a subset of the same stimuli were used in an EEG study, reporting indistinguishable modulation of the power of sensorimotor mu oscillations (which have been linked to motor simulation and the MNS, e.g., Cochin et al., 1999; Arnstein et al., 2011; Press et al., 2011) for human, android and robot actions (Urgen et al., 2013). The present data, however, showed differential modulation of EMG activity for these stimuli. How can we reconcile these findings in the light of the recent experimental evidence? One possibility is that EMG activity does not directly reflect the activity of the premotor cortex, which has been the focus of most prior work. EMG instead partially reflects the activity of the primary motor cortex, and is also susceptible to other influences (see Contributions of EMG: Mechanisms of Action Observation and Imitation).

### Lateralization in Action Imitation and Action Observation

In the present study, during explicit action imitation, EMG activity in the right hand was greater than the activity in the left hand. This is unsurprising given that participants were explicitly asked to imitate the agents’ actions, which were right handed, but assures that EMG can reliably pick up imitation-related activity. More interestingly, in the explicit imitation condition, we found enhanced EMG activity also in the stationary left arm. Furthermore, the EMG activity in the left arm was also present during passive observation; in fact, it was greater than the activity in the right arm.

These results are consistent with previous reports that observation of actions involving one hand can influence motor activity related to both hands of the observer (Borroni and Baldissera, 2008; Borroni et al., 2008). Why did the supposedly passive, non-dominant left arm, show activity during both action imitation and action observation? One possibility is a spatial compatibility effect, whereby observing an action performed on the one side of the screen (here, left) would elicit activity in the same side of the body. Such spatial compatibility effects are well-documented, specifically in studies using stimulus response compatibility paradigms (for a review, see Lu and Proctor, 1995). In fact, it has been suggested that motor resonance may be linked not to the specific arm that performs the action, but to the side of space of the observed action: Kilner et al. (2009) reported that attenuation of beta oscillations during action observation, which show mirror-like properties and are thought to index the activity of primary motor cortex (see Kilner and Frith, 2007 and Contributions of EMG: Mechanisms of Action Observation and Imitation), was greater in the contralateral hemisphere. Greater motor cortex activity in the contralateral side, i.e., the right motor cortex, might then produce greater muscle activity in the left arm.

Another reason for our pattern of findings, especially in the observation condition, could be inhibitory processes that suppress activity of the dominant (right) arm when no action takes place (i.e., during action observation). The presence of inhibitory influences during action observation was recently highlighted (Cross et al., 2013; Vigneswaran et al., 2013). The left arm, on the other hand, could receive less inhibition. Lateralization of premotor and motor cortical processing and the relationship to muscle activity is a complex neuro-computational problem (Baldissera et al., 2001; Fadiga et al., 2005; Churchland et al., 2012; Shenoy et al., 2013). Future studies could examine these differences in arm EMG activity by comparing right and left handers’ reactions to actions performed by right and left arms.

### Sensitivity to Human Motion

In addition to different patterns of lateralization in action observation and imitation, we found that muscle activity for action imitation and observation appeared to be sensitive to the presence of biological motion. That is, EMG responses during explicit imitation as well as observation were greater to an agent that not only looked like a human, but also moved like one. The synchronization results further showed greater linking of participants’ EMG dynamics to human motion. On the one hand, this could be consistent with the idea that MNS is specialized for biological actions (Tai et al., 2004). However, participants were able to faithfully imitate actions produced by all three agents, which along with other studies listed in Section “Artificial Agents in Cognitive Neuroscience,” challenge the notion of strong selectivity. Rather, what these results indicate may be that the nervous system preserves “temporal fidelity” between seen and performed movements even when participants are not instructed to carefully imitate motion trajectory.

The observed greater EMG response to human motion may have several possible sources. On one hand, biological movements have specific dynamics, and are more complex and familiar in the context of human actions. Within the experiment, however, human motion was presented less frequently (where non-human movement was represented by both the Android and Robot conditions and thus was seen twice as often). Thus it is possible imitation of human movements may have involved more attention or effort, which could result in overall increase in muscle tension. A related “affective” explanation may be that viewing a human elicits greater arousal (but note that participants did not rate the human as eliciting more arousal than the other agents, see Supplementary Materials 2.1), which can influence muscle tone and be detected through EMG (Hoehn-Saric et al., 1997). However, we believe such generic accounts are insufficient to account for the effect. Corrugator activity (brow furrowing), an indicator of effort (de Morree and Marcora, 2010), was greater for *Non-Human Motion*, particularly in the observation condition (see Supplementary Materials 2.2.3). If greater effort were associated with correctly imitating human motion, we would expect the opposite pattern. A delay in reaction to human motion could be another potential indicator of effort in the form of a speed-accuracy tradeoff. However, both in an action prediction study (Saygin and Stadler, 2012), and an attentional capture and cueing study (Li et al., 2015) behavioral data were instead modulated by *Non-Human Appearance* (i.e., Robot condition) indicating generic effort or arousal effects are unlikely to underlie the EMG differences in the current study. Rather, we suggest the significant interactions with Time in the data, and the comparisons of cross-correlation lags demonstrate that the results are better viewed as preserved dynamics between perceived movement and executed movement rather than a delay *per se*. This is a much more interesting possibility, is consistent with prior work (Bouquet et al., 2007; Watanabe, 2008), and should be a fruitful direction to explore in future studies of dynamics of imitation of human and non-human movements, ideally with motion capture along with EMG (Thoroughman and Shadmehr, 1999; Casile and Giese, 2006).

### Contributions of EMG: Mechanisms of Action Observation and Imitation

Electromyography can be an important tool for understanding mechanisms underlying action observation and imitation. It is increasingly understood that in addition to MNS, primary motor cortex is also involved not only in imitation but also in action observation (Borroni and Baldissera, 2008; Hari et al., 2014). However, the relationship between the primary motor cortex, premotor cortex, and the peripheral motor system is not yet well-understood. EMG complements methods such as EEG, fMRI and MEG, and by examining actual muscle activity during action observation and imitation, provides an important contribution to the study of action observation and imitation.

Our specific findings pose further interesting questions for the neuroscience community. The data demonstrates that there is muscle activity in the non-dominant arm while the dominant arm is imitating an action, as well as when observing an action performed by the opposite arm. Is this activity related to the dominance of the right arm, the side the action is observed, or to inhibitory neural processes? Further studies that can dissociate effector from spatial compatibility effects such as that of Kilner et al. (2009) could help clarify the underlying reasons.

As for the modulation of EMG by motion dynamics in both observation and imitation, this feature of similarity between the observer and the observed agent might be especially important for imitation, even when it is not explicitly demanded of the participants. In future studies, these can be analyzed with more sophisticated methods and motion capture. Furthermore, the sensitivity of arm EMG to human motion during observation adds a new finding to our multi-modal imaging work with these stimuli, as well as the corresponding research questions regarding the role of human-likeness in action processing. Our previous work did not show any selectivity for biological motion, though there were effects of visual appearance in both behavioral (Saygin and Stadler, 2012; Li et al., 2015) and neuroimaging studies (the extrastriate body area with fMRI and in the frontal theta oscillations in EEG; Saygin et al., 2012; Urgen et al., 2013). Taken together, these studies suggest that EMG taps into processes that we were not able to measure with the brain imaging methods, and adds to efforts to get a more comprehensive picture of the human action processing, MNS and embodiment. Last but not least, since studies have explored EMG in relation to single-cell level activity in motor cortex in non-human primates (Santucci et al., 2005; Kalaska, 2009; Churchland et al., 2012), applying this method to action processing in humans has the potential to help us make better inferences about the physiological mechanisms underlying action imitation and observation, bridge between different methods and brain areas, as well as provide opportunity for exploring cross-species similarities and differences.

### Social Robotics and Artificial Agent Design

Finally, our results have implications for social robotics. One important topic in social robotics today is the design principles of humanoid robots. Neuroscience research can inform how we should design robots that people can seamlessly interact with, as they can with human social partners. In fact, input from cognitive and neural sciences to robotics is essential in this endeavor. In the present study, we found evidence for sensitivity to human motion even during passive observation. Given that unconscious mimicry processes can influence emotional and social processes (Chartrand and Bargh, 1999; Carr et al., 2003), human-robot interaction studies that focus only overt behaviors may miss important implicit effects that may be highly relevant to the identification of design principles for neuro-ergonomic robots.

## Author Contributions

GH, BU, PW, and AS designed the study; AS and BU provided experimental materials; GH programmed and ran the experiment; GH conducted the analyses in consultation with the other authors; GH, BU, PW, and AS wrote the paper.

## Conflict of Interest Statement

The authors declare that the research was conducted in the absence of any commercial or financial relationships that could be construed as a potential conflict of interest.
