# Supplementary material for: Observation and imitation of actions performed by humans, androids, and robots: an EMG study
Source: Front Hum Neurosci. 2015 Jun 19;9:364. doi: 10.3389/fnhum.2015.00364 (PMC4473002; doi:10.3389/fnhum.2015.00364)
Supplement: Supplementary file 1 [file Data_Sheet_1.PDF]

Supplementary Material for Observation and Imitation of Actions Performed by Humans, Androids and Robots: An EMG study

Galit Hofree, Burcu A. Urgen, Piotr Winkielman, & Ayse P. Saygin

1. Supplementary Methods

1.1. Ratings methods

At the beginning of the experiment, participants viewed a video of each agent (Human, Android and Robot), and were asked to rate each agent on how humanlike they found the agent (*Humanlikeness*) and how comfortable the agent made them feel (*Comfort*). Ratings were made on a 9-point Likert scale (1 = not at all, 5 = moderately, 9 = very).

At the end of the observation and imitation conditions (see manuscript), participants were once again asked to rate the agents on *Humanlikeness* and *Comfort*. They were also asked how creepy/scary/weird they found the agent (*Creepiness*). Additional questions included: How arousing/exciting did the agent make them feel (*Arousal*), how happy/good did the agent make them feel (*Positive*), and how sad/bad the agent made them feel (*Negative*). All ratings were made on a 9-point Likert scale. We had participants rate these questions after the experiment so that they would not influence their observation and imitation behavior, which was our main measure of interest.

These ratings were chosen for several reasons. They correspond to previous work in robotics where subjects are asked to rate agents on familiarity, comfort, and eeriness (Bartneck et al., 2009). In addition, previous experiments conducted by the authors with real androids had participants freely describe the agent they viewed. These descriptions included several key terms – words related to humanlikeness, and negative emotional words, including specifically creepy, weird and scary (Hofree et al., 2014). We included measures of affect (arousal, negative affect and positive affect) in order to further examine participants' emotional reactions.

1.2. EMG methods

1.2.1. Calculating the baseline

Unfortunately we discovered that the EMG activity during the time meant for measuring the baseline was very noisy. Participants moved their arms quite a lot although they were instructed to stay still. Using a standard method like the average of the preceding 2000 ms resulted in negative z-scored EMG activity (except in the right arm imitation condition). We therefore explored alternative means of eliciting a clean baseline, and settled on finding the minimum value. More specifically, the baseline was calculated by choosing the minimum value of an interval of 2000 ms before each trial, and then using a sliding window to smooth baseline values over trials.

This different method did not have significant effects on the pattern of the results. A MANOVA including Condition, Arm, Motion and Time (like that found in the manuscript) on the EMG data using a 2000 ms average baseline resulted in a similar set of main effects and interactions, although some effects were dampened because of the noisy baseline. Specifically looking at effects of Motion, we found a Motion x Time interaction ($F(9,234)=3.1$, $p=0.002$, *partial* $\eta^2=0.10$), as well as Condition x Motion x Time ($F(9,234)=3.35$, $p=0.001$, *partial* $\eta^2=0.11$) and Condition x Arm x Motion x Time ($F(9,234)=1.98$, $p=0.04$, *partial* $\eta^2=0.07$) interactions, similar to the findings described in the manuscript.

1.2.2. Consideration of subjects who may have imitated with their left arm

No explicit instructions were given as to which arm to use in the second Imitation block. Although all subjects were right handed, some of them might have used their left arm to imitate the actions in the video. This might explain the effect found in the left arm in the *Imitation* condition. In order to test if this is indeed the case, we set a criterion for removal of subjects who demonstrated reliable imitation in their left arm. We removed any subjects whose left arm EMG activity during the trial in the *Imitation* condition raised more than 0.5 std beyond the measurement at the initial 0-500 ms interval. This threshold was decided on based on EMG measurements in the right arm and in facial EMG in previous experiments, during explicit imitation conditions, which usually peaks well above 1 std. We found 4 subjects that needed to be removed. We ran the main analyses with these subjects removed and found the same pattern of results.

Specifically, we ran a MANOVA with Condition, Arm, Motion and Time as factors, as detailed in the results in the main manuscript. Focusing on effects of Motion, we found a significant Motion x Time interaction ($F(9,198)=32.54$, $p=0.009$, *partial* $\eta^2=0.10$), Arm x Motion interaction ($F(1,22)=10.51$, $p=0.04$, *partial* $\eta^2=0.08$), Condition x Arm x Motion x Time interaction ($F(9,198)=1.98$, $p=0.002$, *partial* $\eta^2=0.10$). Effects and interactions of Arm and Condition were also found to be similar to those in the main analyses. It does not appear that the pattern found in the left arm is associated with particular participants who imitated with their left arm.

2. Supplementary analyses

2.1. Ratings analyses

We ran repeated measures MANOVAs with Human Motion or Human Appearance as a factor. We collapsed across genders since preliminary analyses did not reveal any effects on these measures. We report pre-experiment ratings for Humanlikeness and Comfort attributes, since these did not significantly differ from post-experimental ratings on these same attributes.

2.1.1. Ratings of Human and non-human motion

We compared ratings for the Human video with a mean of ratings for the Android and Robot videos. Participants drew distinctions of *Human* and *Non-Human Motion* across the various rating categories. They rated *Human Motion* significantly higher on Humanlikeness ($M_{\text{Human Motion}}=6.96$, $M_{\text{Non-Human Motion}}=3.48$; $t(26)=15.66$, $p<0.0001$, $d=6.14$), as expected. They also rated *Human Motion* higher on Comfort ($M_{\text{Human Motion}}=8.44$, $M_{\text{Non-Human Motion}}=3.30$; $t(26)=9.79$, $p<0.0001$, $d=3.84$), demonstrating that they were more comfortable with *Human Motion* than *Non-Human Motion*. Creepiness ratings differed as well. Participants found *Human Motion* significantly less creepy than *Non-Human Motion* ($M_{\text{Human Motion}}=2.67$, $M_{\text{Non-Human Motion}}=5.65$; $t(26)=-5.26$, $p<0.0001$, $d=2.06$).

2.1.2. Ratings of human and non-human appearance

Participants also demonstrated sensitivity to Human Appearance in their ratings. They rated the human appearance videos higher on Humanlikeness than videos with *Non-Human Appearance* ($M_{\text{Human Appearance}}$=6.35, $M_{\text{Non-Human Appearance}}$=2.33; $t(26)=13.34$, $p<0.0001$, $d=5.23$), again as expected. In addition, they rated the *Human Appearance* videos higher on Comfort, ($M_{\text{Human Appearance}}$=5.31, $M_{\text{Non-Human Appearance}}$=3.30; $t(26)=5.57$, $p<0.0001$, $d=2.18$), and lower on Creepiness, ($M_{\text{Human Appearance}}$=4.2, $M_{\text{Non-Human Appearance}}$=5.56; $t(26)=2.19$, $p=0.04$, $d=0.86$), than the videos with *Non-Human Appearance*.

2.1.3. Affective ratings

Participants rated both *Human Motion* and *Human Appearance* as significantly more positive than *Non-Human Motion* and *Non-Human Appearance*, respectively (*Motion:* $M_{\text{Human Motion}}$=4.15, $M_{\text{Non-Human Motion}}$=2.43; $t(26)=4.49$, $p<0.0001$, $d=1.76$; *Appearance:* $M_{\text{Human Appearance}}$=3.26, $M_{\text{Non-Human Appearance}}$=2.48; $t(26)=2.38$, $p=0.02$, $d=0.93$). They rated both as significantly less negative, as well (*Motion:* $M_{\text{Human Motion}}$=1.89, $M_{\text{Non-Human Motion}}$=2.76; $t(26)=-2.43$, $p=0.02$, $d=0.95$; *Appearance:* $M_{\text{Human Appearance}}$=2.15, $M_{\text{Non-Human Appearance}}$=3.11; $t(26)=-2.24$, $p=0.03$, $d=0.88$). Arousal ratings did not differ across categories.

2.1.4 Ratings across the three agents

We ran repeated measures MANOVAs with Agent as a within-subjects factor for each attribute. We collapsed across genders since preliminary analyses did not reveal any effects on these measures. We report pre-experiment ratings for Humanlikeness and Comfort attributes, since these did not significantly differ from post-experimental ratings on these same attributes. Participants rated the agents differently on Humanlikeness, as demonstrated by a main effect of Agent ($F(2,52)=146.54$, $p<0.0001$, *partial* $\eta^2=0.85$). As expected, they found the Robot to be less humanlike than the Android ($M_{Robot}=2.33$, $M_{Android}=4.26$; $t(26)=5.51$, $p<0.0001$, $d=2.16$), and the Android to be less humanlike than the Human ($M_{Android}=4.26$, $M_{Human}=8.44$; $t(26)=-10.98$, $p<0.0001$, $d=4.31$). There was a main effect of Agent in ratings of Comfort as well ($F(2,52)=49.61$, $p<0.0001$, *partial* $\eta^2=0.66$). Participants felt significantly more comfortable with the Human ($M_{Human}=6.96$, $M_{Android}=3.67$, $M_{Robot}=3.30$; *planned contrast comparing Human to a mean of Robot and Android*: $F(1,26)=95.88$, $p<0.0001$, *partial* $\eta^2=0.79$), but were equally uncomfortable with the Android and Robot ($t(26)=0.93$,p=n.s.). This pattern is also reflected in Creepiness ratings: they rated both the Android and Robot as moderately creepy ($M_{Android}=5.74$, $M_{Robot}=5.55$), but not different from each other ($t(26)=0.28$, p=n.s.). They found the Human to not be very creepy, both in comparison to the other agents and in respect to the scale itself ($M_{Human}=2.67$; *planned contrast comparing Human to a mean of Robot and Android*: $F(1,26)=27.64$, $p<0.0001$, *partial* $\eta^2=0.52$). Finally, they found the Human more positive (*planned contrast comparing Human to a mean of Robot and Android*: $F(1,26)=20.18$, $p<0.0001$, *partial* $\eta^2=0.44$), and less negative than these agents as well (*planned contrast comparing Human to a mean of Robot and Android*: $F(1,26)= 5.89$, $p=0.02$, *partial* $\eta^2=0.19$).

2.2. Supplementary EMG analyses

2.2.1. Arm EMG MANOVAs across the three agents

We compared EMG activity across the arms using a 2 (Condition) x 2 (Arm) x 3 (Agent) x 10 (Time) design. This MANOVA revealed similar effects as those found when comparing Human and Non-Human Motion, and Human and Non-Human Appearance. First, there was a main effect of Condition ($F(1,25)=70.13, p<0.0001$, *partial* $\eta^2=0.74$), and Condition x Time interaction ($F(9,225)=17.72, p<0.0001$, *partial* $\eta^2=0.42$), as expected. Participants responded more with both arms in the *Imitation* condition, than in the *Observation* condition. There was also a main effect of Arm ($F(1,25)=6.46, p=0.02$, *partial* $\eta^2=0.21$), and an Arm x Time interaction ($F(9,225)=15.12, p<0.0001$, *partial* $\eta^2=0.38$). As can be seen in Supplementary Figure 1, the *Right* arm response is much greater in the *Imitation* condition than any response in the *Observation* condition, but also much greater than the *Left* arm response overall. This is demonstrated in significant Condition x Arm and Condition x Arm x Time interactions (*Condition x Arm:* $F(1,25)=52.52, p<0.0001$, *partial* $\eta^2=0.68$*; Condition x Arm x Time:* $F(9,225)=15.10, p<0.0001$, *partial* $\eta^2=0.38$).

We also found significant modulation of muscle activity by the observed Agent. There was a significant Agent x Time interaction, $F(18,450)=2.72, p<0.0001$, *partial* $\eta^2=0.10$. Participants reacted slower to *Human* stimuli than to the other agents (Supplementary Figure 1). Interestingly, we also found an Arm x Agent interaction ($F(2,50)=6.79, p=0.002$, *partial* $\eta^2=0.21$). Separate MANOVAS for each arm revealed that muscle activity in the *Left* arm was increased for the *Human* condition compared with the *Robot* and *Android* conditions (*marginal main effect of Agent:* $F(1,26)=2.54, p=0.09$, *partial* $\eta^2=0.09$), while muscle activity in the *Right* arm did not differ between the agents (both Agent and Agent x Time not significant). This pattern was also demonstrated in the Omnibus MANOVA by a Condition x Agent x Time interaction ($F(18,450)=1.83, p=0.02$, *partial* $\eta^2=0.07$), as well as a Condition x Arm x Agent x Time interaction ($F(18,450)=1.90, p=0.02$, *partial* $\eta^2=0.07$).

2.2.2. Action type analyses

Previous literature suggests that there might be a neural distinction associated with observation and imitation of transitive (involving an object) and intransitive actions. The monkey MNS appears to only activate in association with transitive actions, while the human MNS is active when observing or executing both types of actions (e.g., Fadiga et al., 1995). In addition, there appears to be a partial dissociation between neural systems involved in production and imitation of these two types of actions: there are apraxias that demonstrate specific deficits in producing transitive but not intransitive actions (Buxbaum et al., 2007). Some suggest that intransitive actions are particularly important since they are involved in human communication and experienced commonly in social situations (Press et al., 2008). Liepelt et al. (2010) demonstrated that this distinction influences motor priming of non-human agents. In their study, they found that motor priming effects did not differ between a human agent and a non-human agent when viewing (still photos of) them preforming transitive or non-communicative intransitive actions. However, when viewing communicative intransitive actions, they found that

human actors produced motor priming effects, and non-human actors did not. Although it is not our main focus of research, our study includes 7 actions that are easily separated into transitive and intransitive actions in order to examine whether this distinction influenced motor activity during observation and imitation of both human and non-human agents. We therefore ran the main MANOVA, and included Transitivity as a factor. We sorted the 7 actions included the following way: drinking, wiping, grasping and holding a paper were all marked Transitive, while nudging, waving and bowing were all marked Intransitive (although note that these are all communicative intransitive gestures).

Results can be found in Supplementary Figure 2. We found that overall arm EMG activity peaked at a greater magnitude for *Intransitive* actions, as is observed in a significant Transitivity x Time interaction ($F(9,234)=9.20$, $p<0.0001$, *partial* $\eta^2=0.26$), and t-test comparisons for the 2.5s and 3s intervals (2.5s: $t(26)=4.19$, $p<0.001$, $d=1.64$; 3s: $t(26)=3.44$, $p=0.002$, $d=1.35$). This effect was magnified in the *Imitation* condition, using the *Right* arm (*Condition x Transitivity x Time interaction:* $F(9,234)=6.2$, $p<0.0001$, *partial* $\eta^2=0.20$; *Arm x Transitivity x Time interaction:* $F(9,234)=8.28$, $p<0.0001$, *partial* $\eta^2=0.24$; *Condition x Arm x Transitivity x Time interaction:* $F(9,234)=5.72$, $p<0.0001$, *partial* $\eta^2=0.18$).

Interestingly we found that Transitivity influenced arm EMG response to the agents differently. We found a marginally significant Agent x Transitivity interaction ($F(2,52)=2.61$, $p=0.08$, *partial* $\eta^2=0.09$), and marginally significant Agent x Transitivity x Time interaction ($F(18,468)=1.53$, $p=0.07$, *partial* $\eta^2=0.06$). Participants produced greater EMG response overall to *Intransitive* actions carried out by the *Human* agent.

However, this influence differed across the arms and conditions, as suggested by a Condition x Arm x Agent x Transitivity interaction ($F(2,52)=3.51$, $p=0.04$, *partial* $\eta^2=0.12$), by an Arm x Agent x Transitivity x Time interaction ($F(9,234)=9.20$, $p<0.0001$, *partial* $\eta^2=0.26$), and by a Condition x Agent x Transitivity x Time interaction ($F(18,468)=2.50$, $p=0.001$, *partial* $\eta^2=0.09$). These differences can be viewed across Supplementary Figure 2.

Separate MANOVAs for the two arms demonstrate that *Left* arm EMG is greater for *Intransitive* actions than *Transitive* actions regardless of Agent and Condition, as is revealed in a main effect of Transitivity ($F(1,26)=4.39$, $p=0.046$, *partial* $\eta^2=0.14$), and a Transitivity x Time interaction ($F(9,234)=2.85$, $p=0.003$, *partial* $\eta^2=0.10$). *Right* arm EMG activity is also influenced by Transitivity, as is demonstrated by a Transitivity x Time interaction ($F(9,234)=11.19$, $p<0.0001$, *partial* $\eta^2=0.3$), and Condition x Transitivity x Time interaction ($F(9,234)=8.29$, $p<0.0001$, *partial* $\eta^2=0.24$). However, it appears that *Right* arm EMG response to the different agents was modulated by Transitivity, as can be seen by a marginally significant Agent x Transitivity interaction ($F(2,25)=3.25$, $p=0.06$, *partial* $\eta^2=0.21$), and by an Agent x Transitivity x Time interaction ($F(18,468)=2.15$, $p=0.004$, *partial* $\eta^2=0.08$ The different effect of *Intransitive* vs. *Transitive* actions is greater for the *Android* and *Human* than the *Robot*. This effect is amplified in the *Imitation* condition, as is demonstrated by a Condition x

Agent x Transitivity interaction ($F(2,52)=4.66$, $p=0.01$, *partial* $\eta^2=0.15$), and by a Condition x Agent x Transitivity x Time interaction ($F(18,468)=2.48$, $p=0.001$, *partial* $\eta^2=0.09$).

These findings demonstrate that arm EMG activity is greater to intransitive actions, during both observation and imitation of these actions. Interestingly, there appears to be greater response specifically to human agents who produce intransitive actions. This corresponds well to the hypothesis that intransitive actions carry special social importance for humans.

2.2.3. Facial EMG analyses

We ran similar analyses to those run on arm EMG. That is, we ran repeated measures MANOVAs with Condition, Motion or Appearance and Time. Analyses with Gender as a factor are reported below in section 2.2.4.

2.2.3.1. Zygomaticus EMG responses

We first ran a MANOVA including Condition, Motion and Time as factors. Zygomaticus EMG responses differed across conditions, as is demonstrated by a main effect of Condition ($F(1,26)=20.18$, $p<0.0001$, *partial* $\eta^2=0.44$), and a significant Condition x Time interaction ($F(1,26)=9.54$, $p<0.0001$, *partial* $\eta^2=0.27$). As can be seen in Supplementary Figure 3 top row, participants smiled more during the *Imitation* condition. In addition, zygomaticus activity demonstrated sensitivity to human motion ($F(1,26)=6.77$, $p=0.02$, *partial* $\eta^2=0.21$). However, this sensitivity was modulated by the condition in which the videos were observed, as demonstrated by a significant Condition x Motion interaction ($F(1,26)=6.48$, $p=0.02$, *partial* $\eta^2=0.20$). Participants smiled more to *Human Motion*, but this differentiation in response was greater during the *Imitation* condition.

We also ran a MANOVA with Appearance as a factor. Besides the effects of Condition (which are similar to those above), there was also a main effect of Appearance, $F(1,26)=4.82$, $p=0.04$, *partial* $\eta^2=0.16$. *Human Appearance* elicited greater zygomaticus response than *Non-Human Appearance*. Overall, these results demonstrate that people smile more to human form and motion, but interestingly this positive response to human motion is modulated by actual imitation of that motion.

2.2.3.2. Corrugator EMG responses

A MANOVA including Condition, Motion and Time revealed interesting results (see Supplementary Figure 3 bottom row). First, corrugator activity differs across conditions, as is demonstrated by a main effect of Condition ($F(1,26)=29.63$, $p<0.0001$, *partial* $\eta^2=0.53$). Participants frown more while observing the videos, than when imitating them. Interestingly, they frown more to *Non-Human Motion*, demonstrated by a main effect of Motion ($F(1,26)=88.46$, $p<0.0001$, *partial* $\eta^2=0.77$), and significant Motion x Time interaction ($F(1,26)=2.05$, $p=0.03$, *partial* $\eta^2=0.07$). This response is modulated by condition, like the zygomaticus, as can be seen in a significant Condition x Motion interaction ($F(1,26)=108.84$, $p<0.0001$, *partial* $\eta^2=0.81$). However, for the corrugator,

sensitivity to *Human Motion* is stronger during the *Observation* condition. Finally, a similar MANOVA with Appearance did not demonstrate any significant effects of *Human Appearance* on corrugator response.

2.2.4. Gender effects in EMG analyses

2.2.4.1. Gender effects in arm EMG responses

We ran analyses with Gender as a between-subjects factor as it has been suggested to play a role in action processing (e.g., Sokolov et al., 2011). The genders differed in their arm EMG responses. We first ran a repeated measures MANOVA with Condition, Arm, Motion, Time, and Gender as factors. *Males* responded earlier than *Females*, as is demonstrated in a significant Time x Gender interaction, $F(9,225)=6.75$, $p<0.0001$, *partial* $\eta^2=0.21$. In addition, Condition influenced the genders differently in their responses. We found significant Condition x Gender and Condition x Time x Gender interactions (*Condition x Gender:* $F(1,25)=5.36$, $p=0.03$, *partial* $\eta^2=0.18$; *Condition x Time x Gender:* $F(9,225)=6.83$, $p<0.0001$, *partial* $\eta^2=0.22$). Although both genders produced stronger responses during the *Imitation* condition, *Males* responded earlier and stronger than *Females*, specifically in the *Imitation* condition. There was also a significant interaction of Arm x Time x Gender ($F(9,225)=5.42$, $p<0.0001$, *partial* $\eta^2=0.18$), a significant Condition x Arm x Gender interaction ($F(1,25)=5.58$, $p=0.04$, *partial* $\eta^2=0.15$), and a significant Condition x Arm x Time x Gender interaction ($F(9,225)=5.29$, $p<0.0001$, *partial* $\eta^2=0.18$). Running separate MANOVAs within each arm demonstrates that *Males* react earlier in the *Imitation* condition both arms (*significant Condition x Time x Gender interaction in the left arm:* $F(9,225)=1.95$, $p=0.04$, *partial* $\eta^2=0.07$; *in the right arm:* $F(9,225)=6.61$, $p<0.0001$, *partial* $\eta^2=0.21$). However, *Females* respond with their *Right* arm during the *Observation* condition. That is, while *Males' Right* arm responses are earlier and greater overall (*significant Time x Gender interaction:* $F(9,225)=6.74$, $p<0.0001$, *partial* $\eta^2=0.21$), *Females' Right* arm responses during *Observation* of the videos is greater than *Males*. This is demonstrated by Condition x Gender interaction ($F(1,25)=5.48$, $p=0.03$, *partial* $\eta^2=0.18$). Interestingly, sensitivity to *Human Motion*, modulated by both arm and condition, was also influenced by gender, as is demonstrated by a Condition x Arm x Motion x Time x Gender interaction, $F(9,225)=2.78$, $p=0.004$, *partial* $\eta^2=0.10$. A MANOVA in the right arm demonstrates more clearly that *Males' Right* arm response during *Imitation* is sensitive to human motion (stronger responses to *Human* vs. *Non-Human Motion*), while *Females* show less sensitivity in their *Right* arm response. Here we found a significant Motion x Time x Gender interaction, $F(9,225)=2.12$, $p=0.02$, *partial* $\eta^2=0.08$.

Overall, males demonstrate a stronger, faster EMG response in their right arm during the *Imitation* condition. This response is sensitive to human motion. Females respond with their right arm during the *Observation* condition more so than males do, albeit these responses are very low in magnitude. We did not find any interactions between Gender and Appearance in a similar MANOVA run with Appearance as a factor. We also did not find gender differences in synchronization with video movements.

2.2.4.2. Gender effects in facial EMG responses

We found gender differences in facial EMG responses as well. In a MANOVA including Motion as a factor, the zygomaticus demonstrated a significant Motion x Gender interaction ($F(1,25)=4.39$, $p=0.05$, *partial* $\eta^2=0.15$). *Males* smile more than *Females* to *Human Motion*, but smile less than *Females* to *Non-Human Motion*. That is, *Males* are more reactive to *Human Motion*, than *Females*. This is qualified by a significant Condition x Motion x Time x Gender interaction, $F(9,225)=4.41$, $p<0.0001$, *partial* $\eta^2=0.15$. Gender differences in reactions to human motions in the zygomaticus are stronger during the *Observation* condition. We find a similar Condition x Appearance x Time x Gender interaction when we ran a MANOVA including Appearance ($F(1,26)=3.94$, $p<0.0001$, *partial* $\eta^2=0.14$). Again, *Males'* zygomaticus response is more sensitive to human appearance, but this heightened sensitivity is specifically found in the *Observation* condition. The corrugator also demonstrates a Motion x Gender interaction, $F(1,25)=5.09$, $p=0.03$, *partial* $\eta^2=0.17$. Here we found that *Females* appear to frown more than *Males* to *Non-Human Motion*.

It is interesting that the genders' affective responses are influenced differentially by human form and motion. Males' positive responses, smiling, are more sensitive to human motion and appearance, while females' negative responses, frowning, are more sensitive to non-human motion.

References

Bartneck, C., Kulić, D., Croft, E., and Zoghbi, S. (2009). Measurement Instruments for the Anthropomorphism, Animacy, Likeability, Perceived Intelligence, and Perceived Safety of Robots. *Int. J. Soc. Robot.* 1, 71–81. doi:10.1007/s12369-008-0001-3.

Buxbaum, L. J., Kyle, K., Grossman, M., and Coslett, B. (2007). Left Inferior Parietal Representations for Skilled Hand-Object Interactions: Evidence from Stroke and Corticobasal Degeneration. *Cortex* 43, 411–423. doi:10.1016/S0010-9452(08)70466-0.

Fadiga, L., Fogassi, L., Pavesi, G., and Rizzolatti, G. (1995). Motor facilitation during action observation: a magnetic stimulation study. *J. Neurophysiol.* 73, 2608–2611.

Liepelt, R., Prinz, W., and Brass, M. (2010). When do we simulate non-human agents? Dissociating communicative and non-communicative actions. *Cognition* 115, 426–434. doi:10.1016/j.cognition.2010.03.003.

Press, C., Bird, G., Walsh, E., and Heyes, C. (2008). Automatic imitation of intransitive actions. *Brain Cogn.* 67, 44–50. doi:10.1016/j.bandc.2007.11.001.

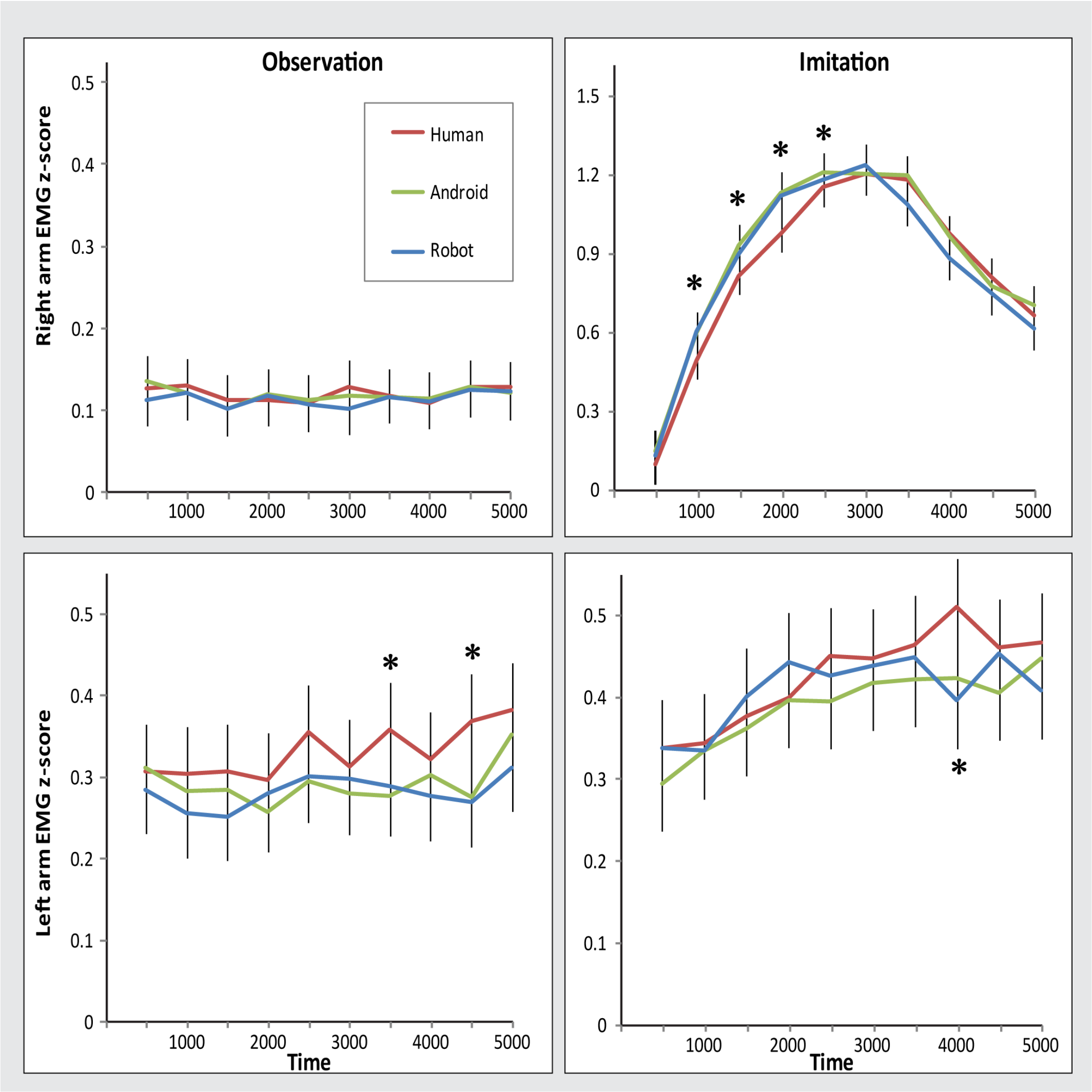

Supplementary Figure 1. EMG response in both arms during observation and imitation of a robot, android and human performing actions with their right arm. **Top**: z-scored EMG activity in the Right arm. **Bottom**: z-scored EMG activity in the Left arm. **Left**: EMG activity during Observation condition. **Right**: EMG activity during Imitation condition. Asterisks denote significance across Motion, at the .05 level.

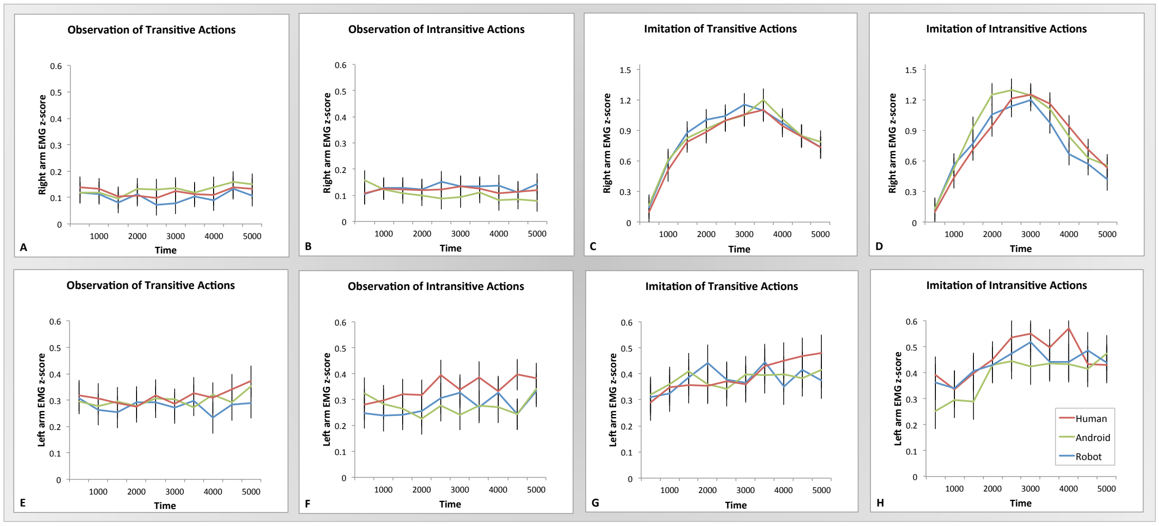

Supplementary Figure 2. EMG response in both arms across conditions and across transitive and intransitive actions. EMG response in both arms is greater to intransitive actions than to transitive actions, and this effect is greater when viewing a human agent. (A) (Right arm) z-scored activity in the right arm during observation of transitive actions. (B) (Right arm) z-scored activity in the right arm during observation of intransitive actions. (C) (Right arm) z-scored activity in the right arm during imitation of transitive actions. (D) (Right arm) z-scored activity in the right arm during imitation of intransitive actions. (E) (Left arm) z-scored activity in the left arm during observation of transitive actions. (F) (Left arm) z-scored activity in the left arm during observation of intransitive actions. (G) (Left arm) z-scored activity in the left arm during imitation of transitive actions. (H) (Left arm) z-scored activity in the left arm during imitation of intransitive actions.

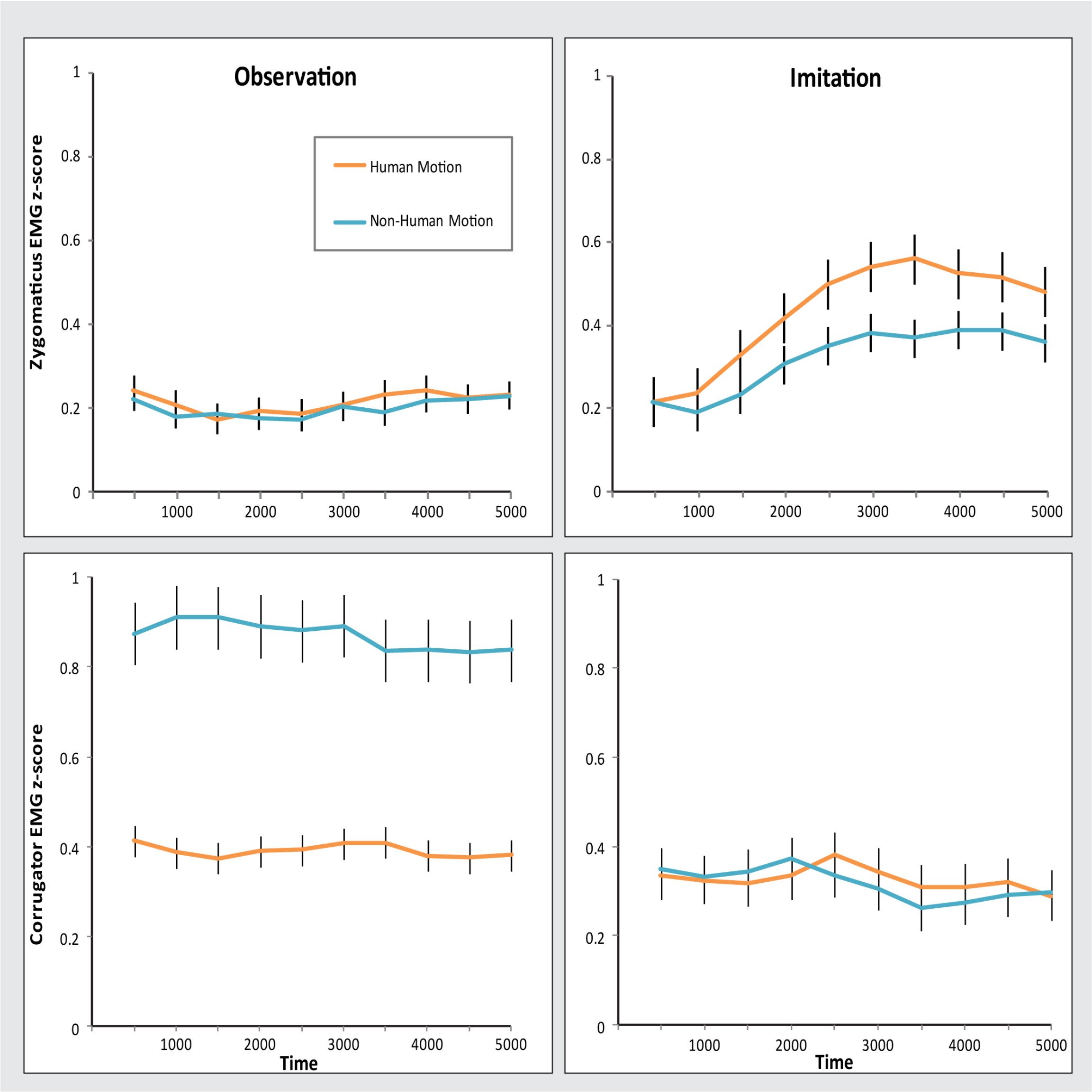

Supplementary Figure 3. Facial EMG response during observation and imitation of human and non-human motion. Top: z-scored zygomaticus EMG activity. **Bottom:** z-scored corrugator EMG activity. **Left:** EMG activity during Observation condition. **Right**: EMG activity during Imitation condition. Post hoc comparisons demonstrate significant differences only for zygomaticus activity in the Imitation condition (from 1500ms onwards significant), and corrugator activity in the Observation condition (all comparisons significant).
